# Kidney Inflammation, Injury and Regeneration

**DOI:** 10.3390/ijms21031164

**Published:** 2020-02-10

**Authors:** Patrick C. Baer, Benjamin Koch, Helmut Geiger

**Affiliations:** Division of Nephrology, Department of Internal Medicine III, University Hospital, Goethe-University, 60596 Frankfurt, Germany; b.koch@med.uni-frankfurt.de (B.K.); h.geiger@em.uni-frankfurt.de (H.G.)

Damage to kidney cells can occur due to a variety of ischemic and toxic insults and leads to inflammation and cell death, which can result in acute kidney injury (AKI). Inflammation plays a key role in the injury of renal cells, as well as subsequent cellular regeneration processes. However, persistent chronic inflammation may trigger renal fibrosis. The investigation of the molecular mechanisms involved in each individual injury is currently insufficiently elucidated. Whereas the kidney has a remarkable capacity for regeneration after injury and may completely recover depending on the type of renal lesions, the options for clinical intervention are restricted to fluid management and extracorporeal kidney support. AKI is still associated with high morbidity and mortality incidence rates, and it also bears an elevated risk of subsequent chronic kidney disease. Therefore, the development of novel therapies to improve renal regeneration capacity after AKI, to preserve renal function, and to prevent AKI is urgently needed. In this context, we wanted to offer a forum for the publication of new results on renal inflammation, injury and regeneration, as well as for the review and discussion of existing studies from this interesting research field.

This Special Issue covers research articles that investigated the molecular mechanisms of inflammation [1,2,3] and injury [4,5] during different renal pathologies and renal regeneration [6], diagnostics using new biomarkers [7,8,9], and the effects of different stimuli like medication or bacterial components on isolated renal cells or in vivo models [10,11,12], all of which were summarized in a very simplified manner. Furthermore, this Special Issue contains important reviews that dealt with the current knowledge of cell death and regeneration [13,14], inflammation [15,16,17,18], and the molecular mechanisms of kidney diseases [19,20,21,22]. In addition, the potential of cell-based therapy approaches that use mesenchymal stromal/stem cells (MSCs) or their derivates is summarized [23,24,25]. This edition is complemented by a series of reviews that deal with the current data situation on other very specific topics like diabetes and diabetic nephropathy [26,27,28], as well as new therapeutic targets [29].

In this Special Issue, twelve original research articles are presented that dealt with different questions and the research models used within. The findings of Mocker and co-workers demonstrate that renal chemerin expression, a chemoattractant adipokine, is associated with processes of inflammation and fibrosis during renal damage [2]. The protection of kidney function by attenuating induced renal inflammation was shown with the use of Farnesiferol B, an agonist of a receptor that is expressed by renal tubular epithelial cells [1]. The xanthin oxidase inhibitor febuxostat is shown to exert anti-inflammatory action and protect against diabetic nephropathy development [3]. Kidney injury leading to focal segmental glomerulosclerosis was shown by variants in the collagen 4A5 gene, demonstrating that the molecular genetics of different players in the glomerular filtration barrier can be used to evaluate the causes of kidney injury [5]. In addition, another study suggested that renal disease in colitis mice might be associated with changes in glomerular collagens and glomerular filtration barrier-related proteins [4].

No injury or inflammatory effects of two anti-diabetically used gliflozins on proximal tubular epithelial cells that were cultured in hyperglycemic conditions were found [10]. Stimulations with bacterial lipopolysaccharide were used to investigate acute renal fibrosis in a model of sepsis-induced AKI [11] and the inflammatory cascade of obese kidney fibrosis in a metabolic endotoxemia mouse model [12].

A very interesting approach investigated the regeneration potential of MSC-derived extracellular vesicles that were transfected with specific miRNA mimics [6]. Furthermore, others introduced a kidney injury test for the noninvasive monitoring of IgA nephropathy progression [9]. Schiffer and co-workers described CXCL13 blood levels as a biomarker in T-cell-mediated rejection [8]. The marker correlates with B-cell involvement and might help to identify patients with a more severe clinical course of rejection [8]. Others demonstrated that that specific IL-18 genotypes may play a role in the etiology and progression of renal cell carcinoma and serve as useful early detection biomarkers.

Priante and co-workers reviewed the different modalities of apoptosis, necrosis, and regulated necrosis in kidney injuries in order to find evidence for the role of cell death, which may pave the way for new therapeutic opportunities [14]. Others discussed the molecular basis of injury and repair in distinct cell types of the kidney during arterial hypertension [21]. In this context, the main mechanisms of kidney regeneration, while focusing on epithelial cell dedifferentiation and the activation of progenitor cells with special attention on the potential niches of kidney progenitor cells, were also lighted [13]. Three reviews by Yun [25], Bochon [23] and Lee [24] summarized the therapeutic potential and efficacy of MSCs, which are primarily associated with their capability to inhibit inflammation and initiate renal regeneration. MSCs predominantly act through secreted factors, including microRNAs that are contained within extracellular vesicles, cytoprotective effects anti-inflammatory effects, anti-apoptotic effects, and the suppression of oxidative stress. In addition, further reviews summarized the inflammation-mediated mechanisms or the inflammasome in various renal diseases [15,16,17,18,26,27]. Very interesting and new approaches shed a light on the role of non-coding RNAs, either in the progression of glomerular or tubulointerstitial kidney diseases [20] or as new therapeutic targets or biomarkers for fibrotic changes [29]. Another interesting work reviewed the involvement of salt-inducible signal transduction pathways in AKI and discussed the possibility of new therapy options [22].
